# A Low Density Microarray Method for the Identification of Human Papillomavirus Type 18 Variants

**DOI:** 10.3390/s131012975

**Published:** 2013-09-26

**Authors:** Thuluz Meza-Menchaca, John Williams, Rocío B. Rodríguez-Estrada, Aracely García-Bravo, Ángel Ramos-Ligonio, Aracely López-Monteon, Rossana C. Zepeda

**Affiliations:** 1 Laboratory of Molecular Biology, Health Services Studies Centre, University of Veracruz, 147 Carmen Serdan St., Centre, Veracruz-Llave, Veracruz 91700, Mexico; E-Mails: xio_estrada@hotmail.com (R.B.R.-E.); ahhhra_gabr@hotmail.com (A.G.-B.); 2 Biomedical Research Centre, University of Veracruz, Av. Luis Castelazo Ayala St. Xalapa-Enriquez, Veracruz 91120, Mexico; E-Mails: angramos@uv.mx (Á.R.L.); aralopez@uv.mx (A.L.-M.); rossanazepeda@gmail.com (R.C.Z.); 3 LADISER Inmunology and Molecular Biology, Faculty of Chemical Sciences, University of Veracruz, Orizaba, Veracruz 94340, Mexico; 4 Department of Biochemistry, Biosciences Institute, University College Cork, College Road, Cork, Ireland; E-Mail: whostheguy@gmail.com

**Keywords:** HPV-18, microarray, variants, LCR

## Abstract

We describe a novel microarray based-method for the screening of oncogenic human papillomavirus 18 (HPV-18) molecular variants. Due to the fact that sequencing methodology may underestimate samples containing more than one variant we designed a specific and sensitive stacking DNA hybridization assay. This technology can be used to discriminate between three possible phylogenetic branches of HPV-18. Probes were attached covalently on glass slides and hybridized with single-stranded DNA targets. Prior to hybridization with the probes, the target strands were pre-annealed with the three auxiliary contiguous oligonucleotides flanking the target sequences. Screening HPV-18 positive cell lines and cervical samples were used to evaluate the performance of this HPV DNA microarray. Our results demonstrate that the HPV-18's variants hybridized specifically to probes, with no detection of unspecific signals. Specific probes successfully reveal detectable point mutations in these variants. The present DNA oligoarray system can be used as a reliable, sensitive and specific method for HPV-18 variant screening. Furthermore, this simple assay allows the use of inexpensive equipment, making it accessible in resource-poor settings.

## 1. Introduction

Human Papilloma Virus (HPV) has been commonly observed to infect humans. More than 50 different types of HPVs are known to infect the female genital tract and some of these are known to have oncogenic potential [1,2]. The fact that more than 99% of all cervical carcinomas (CCs) are positive for infection with oncogenic HPV types indicates that this is a major etiological factor in the oncogenesis of this neoplasm, as well as in its precursor lesions [3]. Uterine CCs represent the second highest cause of cancer-related deaths among the female population worldwide [4]. HPV-18 is not only a high recurrence and high risk type upon detection [5,6], but after developing CC, it is closely associated with the worst prognosis of all the HPV types [7–9]. HPV-18 is the second most common cause of CC, after HPV-16, with a higher mortality rate [10].

Analysis of genomic polymorphisms among different viral isolates allows the understanding of infection pathways in human populations, as well as studying viral evolution. Studies using DNA sequencing analysis of hundreds HPV isolates from clinical specimens and CC derived cell lines have shown considerable intratypical diversity for HPV-18 phylogenetic lineages [11]. Worldwide studies have shown the existence of polymorphisms within each HPV types known as HPV variants. It has been determined that HPV-16 and HPV-18 have spread during evolutionary human time-span [11–14]. Molecular variants of a given strain can have a change in its nucleotide sequence by at least 2.0% in coding regions and 5% in non-coding regions [15]. Subsequent work has demonstrated that HPV-18 displays its highest polymorphic diversity at the Long Coding Region (LCR), which allows differentiating into lineages resulting in three major phylogenetic clusters [16]. Molecular HPV-18 variants consist of Asian-Amerindian (A-A), African (AF), and European (EU) branches.

Interestingly, a direct correlation between HPV-18 intratypic variants has shown that the type of variant affects the oncological predisposition [17], as well as in others HPV types [18,19]. Analysis of HPV-18 variants has shown that the A-A variant potentially enhances oncogenicity, whereas the EU variant seemed to be associated with a lower risk for CC development [20,21]. Therefore, the detection of intratypic variants may be crucial to assess the risk and provides an appropriate CC prognostic strategy [22]. Based on this, we report a novel approach to detect single nucleotide polymorphisms to distinguish the three possible variants for HPV-18.

## 2. Materials and Methods

### 2.1. HPV-18 Variant Alignments

Alignments were produced using the ClustalW-online multiple sequence alignment tool from EMBL-EBI [23].

### 2.2. HPV-18 Nucleotide Database Search

For the purpose of this technological application, we simplified using a shorter format with only four SNPs (C7486T, C7496G, C7529A and T7530C) to cover the three possible phylogenetic derivatives. HPV-18 DNA sequences were retrieved according to the HPV International Consortium (1997) and used to design probes, stacking oligonucleotides, synthetic targets (stDNAs) and PCR primers. Each HPV-18 variant was obtained by annotations made previously, including probes and synthetic oligonucleotide targets [11,16,17,20].

### 2.3. Oligonucleotide Design

**Figure 1.**
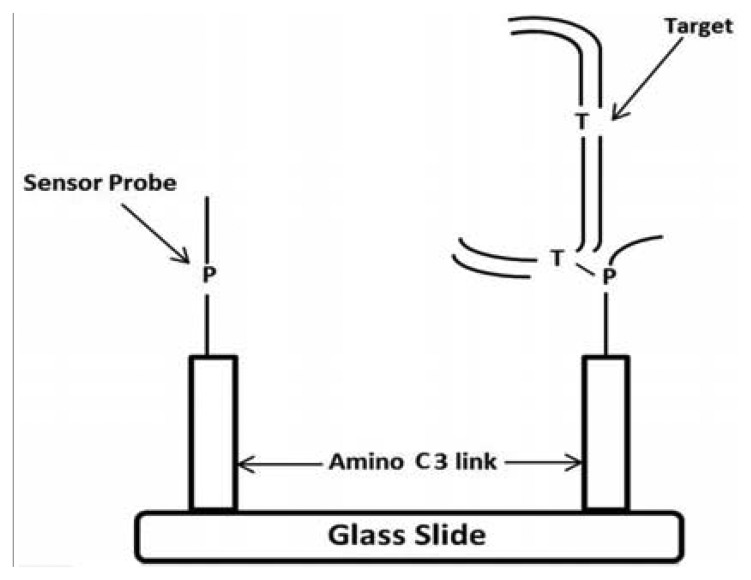
Schematic of the oligoarray system for the detection of A-A, EU and AF variants of LCR. On left side, the sensor probe is depicted without target, while on the right side the DNA target, being previously preannealed stabilizers with the of labeled synthetic DNA, is hybridized against the probe.

PCR primers were designed to amplify a DNA fragment of 137 base pairs (bp) (Forward primer located at 7404–7423 nucleotides [nt] and Reverse primer at 7536–7555 nt) within the HPV-18 LCR (Table 1). To analyze the 7486, 7496 and 7529/7530 sites, two sets of primers were designed for single strand amplification at nt 7498–7520 and 7513–7521, respectively. Stacking oligonucleotides were hybridized to their corresponding DNA targets (either synthetic or biological samples), to produce partially duplex DNAs harboring a 7–9 nt gap, which corresponds to the sequence matched by the capture probes. The model for the detection of EU7486, EU7529, AF7496, AF7530, AA7486, AA7496, and AA7529/7530 base substitutions in LCR HPV-18 that differentiate A-A, EU and AF variants. As shown in Figure 1 the oligoarray was composed by, seven capture probes with a 3′-aminopropanol C3 link fixed on the glass slides. On left side, the sensor probe is depicted without target, while on the right side the DNA target, being previously preannealed stabilizers with the of labeled synthetic DNA, is hybridized against the probe. A total of three capture probes were designed, comprising of one for each possible HPV-18 variant (Table 1). The capture probes, including the 3′-aminolink (3′-aminopropanol) modification for covalent attachment to the glass slide surface [24–26], were purchased from Integrated DNA Technologies Inc. (Coralville, IA, USA).

### 2.4. HPV-18 Oligoarray Design

Initial work consisted of identifying potential capture probes containing the natural polymorphism that corresponds to the introduced blank target at the middle of its sequence. Each of the three variants was targeted by four different single nucleotide sites at the LCR region. Available HPV DNA variant sequences were separately and systematically analyzed using software to simulate virtual hybridization for predicting convenient probes of 7/8/9-mers. We measured values pertaining to thermodynamic stability, depending on the nucleotide sequence and other factors, such as chain length, concentration and presence of ions, nucleic acid concentration of probes, potential secondary structure and similarity properties of DNA sequences [27]. This was performed to allow the discrimination by simultaneous assays. We followed the largest −ΔG value with 100% base pairing, and a lesser value among the wild type probe *vs.* variant target, or vice versa, the variant probe *vs.* the wild type target.

Before analyzing CC samples, stDNAs were employed to optimize this technique. The general design, all capture probes, auxiliary oligonucleotides (AUX), ST, and PCR primer sequences employed are summarized in Table 1.

To optimize the assay, target oligonucleotides with identical sequences to the ones present at A-A, AF, and EU phylogenetic branches were used. Seven stacking oligonucleotides were annealed to their corresponding target DNAs, producing partially duplexed DNA containing a gap composed of the single strand sequence that may hybridize with the capture probes. Seven capture probes were designed, one of which was used to detect the wild type sequence (Table 1). According to previous reports [28], short oligonucleotides with base changes located centrally were selected as probes previously expected to yield good discrimination of point mutations by tandem hybridization at room temperature.

**Figure 2.**
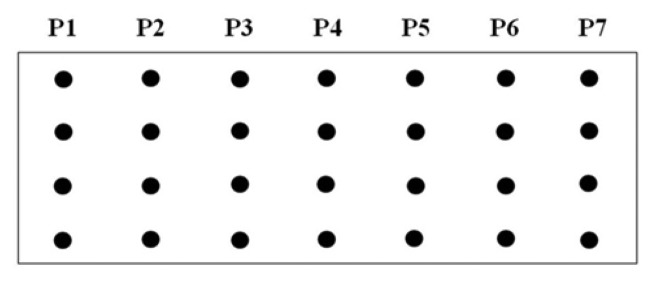
Schematic depicting the DNA capture probe grid across the slide from probe one to seven (P1–P7) displaying four consecutive repetitions for each probe. In order of appearance, for P1 (EU7486), P2 (EU7529), P3 (AF7496), P4 (AF7530), P5 (AA7486), P6 (AA7496), and P7 (AA7529/7530).

A procedure for tethering oligonucleotides to underivatized glass surfaces as a support matrix was employed. Clean glass microscope slides were soaked in 1 N HNO_3_ and absolute ethanol for 30 min each, then rinsed in H_2_O. The slides were dried for 4 h at 90 °C. Oligonucleotide capture probes containing 3′-terminal amino modifications were dissolved in H_2_O to a final concentration of 40 μM and 400 ηL droplets of each probe were applied to the epoxysilanized glass slides in a drop wise manner. As shown in Figure 2, each probe was arranged in quadruplicate in a downward direction from probe one to seven (P1 to P7) and slides were also divided into two groups to test reproducibility. The slides were placed in a high-humidity chamber with a H_2_O reservoir for 2 h at 20 °C. Slides were washed in deionized H_2_O, and air-dried.

### 2.5. Cell Line Targets

Cell lines harboring distinctive punctual mutations from each phylogenetic branch were tested to optimize this array. HPV-18 positive cell lines were obtained, each one containing known mutations present corresponding to each phylogenetic branch: B18-3 cells were used to recognize A-A mutation; HeLa cells and T18-3 cells were used in the hybridization array optimization to detect EU and AF variants, respectively. A total of thirty two CC HPV-18 positive samples were tested. For each case, the presence of HPV-18 was confirmed by using DNA automated sequencing and subsequently the sequences were compared using the basic alignment detection tool [26] to determine the HPV type.

### 2.6. PCR Amplification and Single Strand PCR

Genomic DNA isolation and purification was performed using the Genomic DNA Extraction Kit (Life Technologies Inc., Gaithersburg, MD, USA) according to manufacturer's protocol. DNA concentration, purity and integrity levels were calculated by measuring absorbance at 260 nm in a MBA 2000 (Perkin-Elmer, Waltham, MA, USA) spectrophotometer. The PicoGreen^®^ dsDNA Quantitation Kit (Molecular Probes) was used to quantify the DNA. Reactions contained 0.5 M of each dNTP, 50 mM KCl, 10 mM Tris-HCl (pH 8.4), 1.5 mM MgCl_2_, 1 μM of each primer, 2.5 U of Taq DNA polymerase (Promega, Madison, WI, USA) and purified template DNA (50–100 ηg) in a final volume of 100 μL.

The PCR profile comprised an initial heating step at 94 °C for 5 min, followed by 30 cycles of 94 °C for 30 s, 60 °C for 30 s, and 72 °C for 30 s, with a final extension step at 72 °C for 7 min in a programmable thermal cycler (Gene Amp PCR System 9700; Applied Biosystems, San Francisco, CA, USA). PCR products were assessed by electrophoresis in 2% agarose gel stained with ethidium bromide. Single-stranded target DNA was prepared by cycle synthesis as follows. A 30 μL aliquot of PCR product was processed using Ultrafree (Millipore, Bedford, MA, USA) spin filters (30,000 Mw cutoff), and suspended in the same volume of HPLC-grade H_2_O (OMNI SOLV^®^, EM Science, Charlotte, NC, USA). A 5 μL aliquot of this solution was added to a 100 μL PCR reaction, along with the primer corresponding to the target strand, and was incubated for 40 cycles using the sense oligonucleotide in the next cycle reaction: 94 °C for 5 min; 40 cycles of 94 °C for 30 s; 60 °C for 30 s; 72 °C for 1 min. PCR products were purified before and after re-amplification using the Qiagen Gel Extraction Kit (Edge Biosystems Gaithersburg, MD, USA).

**Figure 3.**
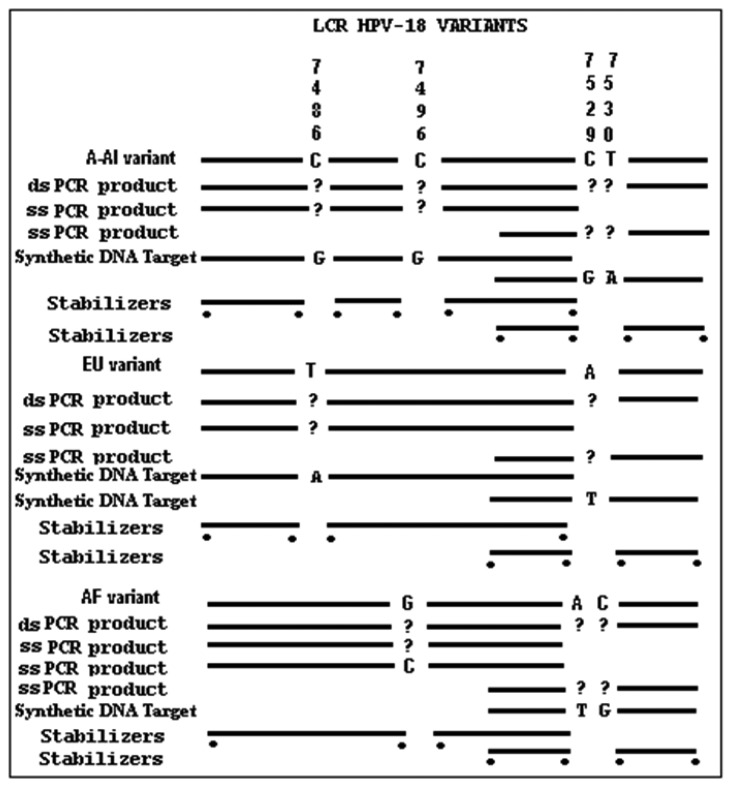
General design of the microarray strategy illustrating composition of phylogenetic variants, plus double strand and single strand PCR products; stDNA, and stabilizers within the HPV-18, LCR. Far left: Type of nucleic acid; Top: Nucleotide Polymorphism interrogated; Dots below lines: Radiolabel P^32^.

In order to synthesize the single strand PCR (ssPCR) product, a second PCR was performed by using only one primer and the PCR product as a template. The procedure was performed as follows. A 20 μL aliquot was processed through Ultrafree (Millipore) spin filters (30,000 Mr cut-off) and dissolved in the same volume of MQ H_2_O. A 3 μL fraction was added to a 50 μL single strand PCR reaction using only the primer that flanks the target region (Figure 3). Reactions were incubated for 27 cycles at 65 °C as stated by Superscript II Reverse Transcriptase instructions (Invitrogen, Carlsbad, CA, USA).

**Figure 4.**
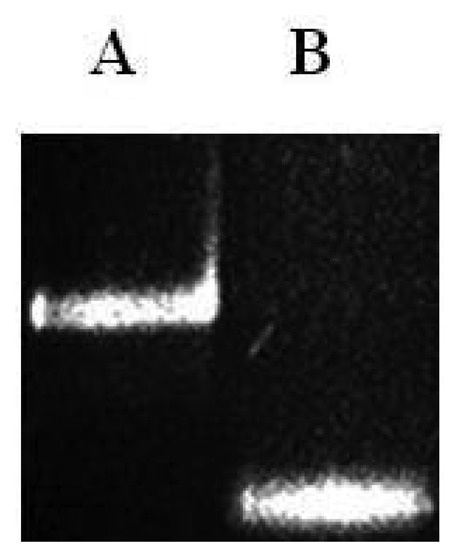
Polyacrylamide gel (7.5%). Lane A: partial duplex containing hybridized stDNAs and stabilizers. Lane B: stDNAs without stabilizers.

As shown in Figure 4 the final ssPCR product was confirmed by comparing this product with the initial double stranded PCR product by mobility shift using agarose gel electrophoresis, partiall duplex runned slower than with no stabilizers. Observable difference in mobility under the electrophoretic pattern occurred due to presence or absence of hybridized stabilizers. To validate microarray recognition patterns, each PCR product from the control and CC samples were sequenced using automated ABI sequencer 337 (Data not shown).

### 2.7. Oligoarray Standardization

Labeling and Pre-Annealing of the Synthetic Auxiliary Oligonucleotides to Target Strands

Six stacking oligonucleotides were 5′-labeled with T4 polynucleotide kinase (Invitrogen). 30 ρmol of each dephosphorylated stacking oligonucleotide; 5X Forward Reaction Buffer (5 μL); T4 Polynucleotide Kinase (10 unit); γ^32^ P-ATP (10 μCi/μL, 7000Ci/mmol); 2.5 μL (NEN, Boston, MA, USA), specific activity 7000 Ci/mMol, and were diluted with sterile H_2_O to 7 μCi/μL, and brought to 25 μL with autoclaved HPLC-grade H_2_O. The reaction mix was incubated at 37 °C for 10 min and the reaction was stopped by adding 5 mM EDTA (2.5 μL).

In the case of the partial duplex target DNA's, a pair of ^32^P-labeled stacking oligonucleotides were pre-annealed with the respective target, either a synthetic DNA or single-stranded PCR product previously synthesized to form a 7-nt gap in each target. The annealed reaction mix contains 50 μL 20X SSC, 10 μL 1M Tris-HCl (pH 8.0), 3 μL 0.5M EDTA, 0.2–12 ρmol of each labeled stacking oligonucleotide, and 20–100 ρM of stDNA or single-stranded DNA target, and HPLC-grade H_2_O to a final volume of 90 μL. The annealing mixture was incubated at 95 °C for 10 min, at 45 °C for 5 min, then 4 °C for 5 min. Unincorporated [γ^32^P]-ATP molecules were removed by filtration using Ultrafree Spin Filters (3000 Mr cutoff) and Microcon filters (Millipore), retained DNA was then dissolved in 20 μL 1X SSC. AUX3 and AUX5 stacking oligonucleotides were 5′-labeled with T4 polynucleotide kinase (Invitrogen, Carlsbad, CA, USA) as described above. Product formation was determined by running the amplicons through a 10.5% polyacrylamide gel stained with ethidium bromide.

In order to standardize\optimize the conditions for this assay, as well as to provide confidence in the interpretation of the signals produced by human samples, reference hybridization patterns were set up using three stDNAs (58 nt), representing the wild type and mutant sequences used as targets. Confirmation of mismatch discrimination in simultaneous assays was tested with capture probes.

### 2.8. Microarray Hybridization

Before hybridization, slides were blocked by soaking for 1 h at room temperature with a solution comprised of 10 mM tripolyphophate, washed with H_2_O and then air-dried [24]. This attachment procedure resulted in a surface density of oligonucleotides attached to glass using the 3′-aminopropanol method (10^10^–10^11^ molecules/mm^2^), which corresponds to intermolecular spacing of about 30–100 Å across the surface [25,26]. The hybridization mix contained: 100 μL 0.5 M tetramethyl-ammonium chloride (TMAC) (Life Technologies, Carlsbad, CA, USA), 9 μL 10 mM Tris-HCl (pH 8.0) (Life Technologies), 0.72 μL 0.5 M EDTA, 1.8 μL 20% (w/v) sodium dodecyl sulfate, 14.4 μL 40% (w/v) polyethylene glycol, 20 μL (10 ρmol) of partial duplex labeled target DNA in 1X SSC, and 35.3 μL HPLC-grade H_2_O. A 40-μL aliquot of this mix was placed on each array and subsequently covered with a cover slip. Slides were incubated in a humid chamber for 3 h at 25 °C. In order to detect the signal, the slides were washed several times in hybridization solution without polyethylene glycol or DNA, air-dried, and then wrapped in parafilm and placed against X-ray film (Kodak BioMax) for autoradiography. Detection of ^32^P-labeled target molecules bound across the hybridization array was performed with a scanner (HP Scanjet 4400c), and analysis of densitometric images was performed by using Scan Analyze 2 software (Stanford University, Stanford, CA, USA). In order to validate results obtained with a different method, PCR products obtained were purified using the QIAEX II kit (QIAGEN Inc., Valencia, CA, USA). Sequencing of each PCR product was performed using the Big Dye Terminator Kit (Perkin-Elmer) and a model 373 automated DNA sequencer.

A total of 45 HPV-18 positive samples were selected: 11 from cytological smears, and 34 from fresh tissues. HeLa cells were used as positive control. The reference sequence was the HPV-18 genome clone (wt HPV-18) (HPV Sequence Databases: PV types and host, Los Alamos National Laboratory Bioscience Division, University of California, Santa Barbara, CA, USA (http://www.stdgen.lanl.gov/). In each case, HPV-18 type was confirmed by using DNA automated sequencing and the BLAST alignment tool (http://blast.ncbi.nlm.nih.gov/Blast.cgi) for sequence comparison in order to determine the viral type.

## 3. Results and Discussion

### 3.1. Phylogenetic Analysis of HPV-18 Variants

HPV evolutionary dispersion pattern has been thoroughly established and is considered to be as old as the human race. Recombination between viral genomes is either non-existent or a very rare event [13,29]. HPV-18 is the most aggressive type out of all known forms of HPVs and the second most frequent. Papillomaviruses are largely dependent upon the host cellular machinery and the use of host DNA polymerase for replication of their genomes. Also, differences in oncogenicity have been reported geographically and ethnically [11,30,31]. Because of this, it is reasonable to employ this variant typing assay for both clinical and research analysis.

Differences in the LCR that impact P105 promoter activity could be responsible for the observed variability in oncogenic potential [20]. However, more studies are needed to clarify the process between involving the genomic phylogenetic variation and the oncogenic development in a certain CC histological type. The hybridization discriminate between WT and variants at this site using oligonucleotide array hybridization ranged from very good to poor, depending on the number and location of mismatches between probe and target. The optimal mismatch discrimination within among probes and targets was identified that occur at room temperature.

Previous studies have demonstrated that HPV-18 specifically diverges into three phylogenetic branches. This can help explain how each variant of a HPV type can have different biological behaviours and being clinically more aggressive than the others HPVs. Differential distribution of HPV-18 variants in cervical lesions supports the hypothesis of a correlation between HPV variant type and CC development [30]. For example, HPV-18 EU variant was almost exclusively found in invasive lesions; while the HPV-18 AF variant is more predominant in normal or pre-invasive lesions. While only in invasive squamous tumours, the A-A variant was found [31].

**Figure 5.**
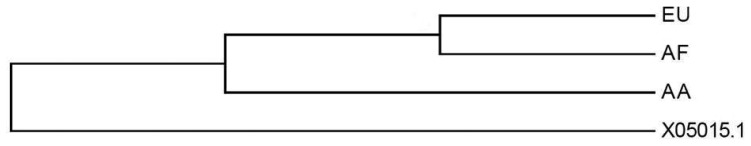
Phylogenetic tree comparing A-A, AF and EU variants similarity at the LCR region.

The evolutionary history was determined using the Neighbour-Joining method [32]. The bootstrap consensus tree inferred from 1,000 replicates is taken to represent the evolutionary history of the taxa analyzed [33]. Branches corresponding to partitions reproduced in less than 50% bootstrap replicates are collapsed. The percentage of replicate trees in which the associated taxa clustered together in the bootstrap test (1,000 replicates) is shown next to the branches. The evolutionary distances were computed using the Maximum Composite Likelihood method [34] and are in the units of the number of base substitutions per site (Figure 5). The analysis involved 4 nucleotide sequences. All ambiguous positions were removed for each sequence pair. There were a total of 7,857 positions in the final dataset. Evolutionary analyses were conducted in MEGA5 [35]. As shown in Figure 4 the analysis resulted on the differentiation among phylogenetic branches. Interestingly, from a phylogenetic perspective the EU and AF variants seem to be closely related than the A-A counterpart. This may corroborate that these two populations were geographically closer and perhaps more interrelated than de Asian-Amerindian group.

### 3.2. Microarray Design

In this new approach, we designed an assay to identify the three major phylogenetic variants simultaneously, based on contiguous SNP by following the DNA microarray principles. Each of the variants in the same 8 nt target were detected very efficiently using Genosensor technology, which required a short target and probe. In addition, it prevents possible secondary structures, which are an important limitation in the DNA array hybridization process. The inclusion of the auxiliary oligonucleotides to create partial duplexes, which covers up possible redundant binding sites in the target strand, is another convenient element of this technology.

The microarray spot pattern was designed as a matrix of consecutive lines made by four identical repetitions, displayed as vertical lines of spots on the glass slide. Each vertical line contains a different probe and each spot contained the same concentration and type of probe. This assay, as with the other hybridization experiments, was performed multiple times to demonstrate the reproducibility of results. Specific probes AA7486, AA7496 and AA7529/7530 were designed for the base paring and recognition of wild type which belongs to the A-A variant. For recognizing the EU variant specific probes were designed named as EU7486 and EU7529. Finally, to be able to detect the sequence target containing the punctual mutation that corresponds to the African variant we used probes AF7496 and AF7530.

### 3.3. Hybridization of Synthetic Oligonucleotide Targets with the Microarray

To test the viability, standardizing and optimal discrimination of mismatches, hybridization experiments involving stDNAs were performed. The glass-tethered 3′-aminolink adjacent to the stacking oligonucleotides probes, were hybridized to partial duplexes composed by stDNAs and (^32^P-labeled) AUX-5 and AUX-3. The process of standardization was followed with three different stDNAs (Figure 3).

Prior to the hybridization with the probes, the two auxiliary oligonucleotides AUX5 and AUX3 (previously ^32^P-labeled) were hybridized into unlabeled stDNAs producing a partial double strand band. To establish if the single stranded target DNA has been annealed to the auxiliary oligonucleotides, annealing reactions were performed. Then, as stated for Figure 3 the presence of pre-annealing/labeling was validated by running the products of pre-annealing and the single chain in a 7.5% polyacrlamide gel. As shown in Figure 3, the synthetic single chain (lane B) has greater mobility than the partial duplex (lane A). In lane B, only a single band was visible corroborating the correct integration of auxiliary fragments to the single strand chain. Next, the double partial duplex was purified from the possible residues of unincorporated auxiliary oligonucleotides. The patterns of hybridization for the stDNAs captured had shown the specificity and selectivity which corresponds to each of their complementary target sequences. As shown, discrimination mismatches are exclusive from their complementary sequence and no unspecific signals were observed (Figure 5). At room temperature, significant hybridization occurred only with the 7-mer and 8-mer probes containing a perfect match at the junction with the stacking oligonucleotides. As expected, our targets were hybridized with the capture probe and assessed using 7 basic combinations of probes; EU7486, EU7529, AF7496, AF7530, AA7486, AA7496 and AA7529/7530 against their complementary stDNA. As shown in Figure 5 stDNA hybridization signals were as follows: (A) displays the matching of the stDNAs and P1 that detects the A-A sequence at nt 7486; (B) Hybridization between stDNAs and P2, which contains the polymorphism that detects the A-A sequence which contains a change at nt 7496; (C) Hybridization signal corresponding to P3 that recognizes the EU sequence at nt 7486; (D) Hybridization signal corresponding to EU7529. (E) Hybridization signal corresponding to P5 at position 7496; (F) Hybridization signal of the synthetic DNA corresponding to P6 and the sequence that belongs to the African variant AF7530; (G) Hybridization pattern detecting the complementary sequence of probe P7. All labeled targets hybridized with their complementary probes, and subsequently were used as positive controls. Equal volumes of each stDNA were tested in a single probe array, mimicking conditions of a patient with multiple HPV-18 variant infection. Hybridization pattern signals for each sample were comparable to the signal pattern from the single target chain variant infection, resulting in no observable unspecific background; hence, the probe capture test is highly specific to the complementary sequence target.

### 3.4. PCR Amplification and Cell Lines Target Preparation for the Hybridization

**Figure 6.**
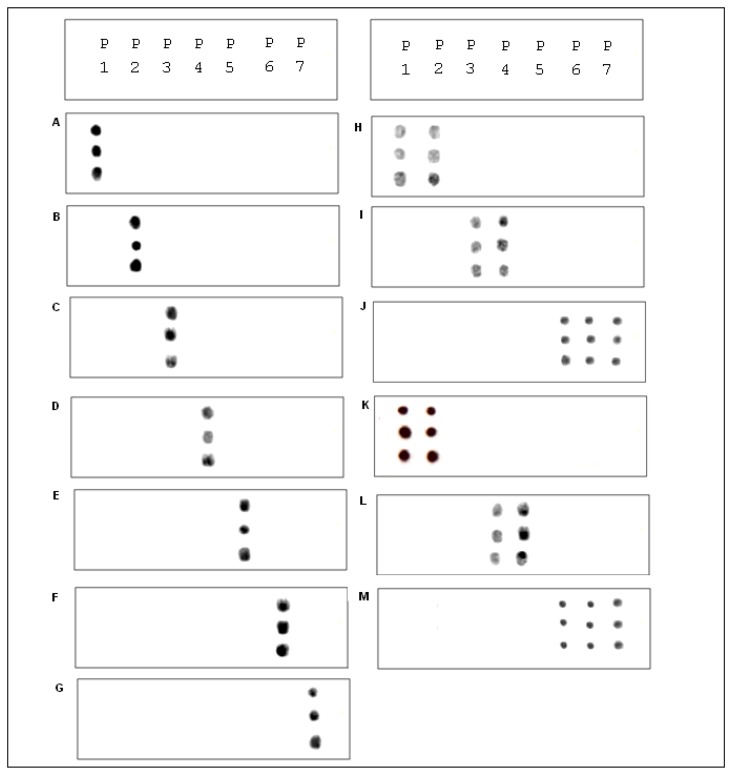
The same set of probes were used on each hybridization pattern of stDNAs (**A**–**G**), cell lines (**H**–**J**) and CC samples (**K**–**M**) explained in the text.

Under the standardization process, the concentrations were adjusted to find the optimal concentration of the DNA sample. It was found that a concentration of 10 ρmol was optimum. The microarray hybridization signals between the cell lines and the capture probe were visualized on autoradiography through previous exposure on radiographic plates overnight (Figure 6H–J). A total of seven rows of hybridization have shown reproducible results. Detection and discrimination among the probes was specific for the recognition site at the stage of standardizing experiment by hybridizing stDNAs. As shown in Figure 5, cell lines were tested as follows: (H) Hybridization pattern for the cell line B18-3 to the variant A-A at nt 7529/7530; (I) Hybridization pattern of HeLa that corresponds to EU; (J) Hybridization pattern of T18-3 that corresponds to AF.

**Figure 7.**
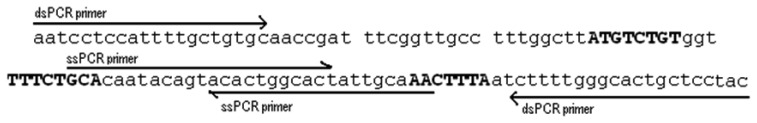
HPV-18 LCR nucleotide sequence showing primers sites with arrows (ds = double strand, ss = single strand) and target regions in bold and uppercase characters.

A set of four primers was designed within the LCR region to amplify the sequence of interest. As shown in Figure 7 an amplicon of 137 bp was obtained flanked by the arrows indicating the sense of polymerization either for double or single stranded amplification. The fragment shown had the right length suitable for hybridization with the oligonucleotide array construct. The analysis was done specifically to include one of each sample related to the three phylogenetic branches: A-A, EU and AF. PCR amplifications were performed to obtain a 137 bp product for each of the cell lines tested on the microarray, and were verified with gel electrophoresis in conjunction with a positive control of HPV-18 LCR. These amplified products, which contain the DNA target chain, were subjected to a second re-amplification by single strand polymerase chain reaction (ssPCR) yielding the single strand target needed for the hybridization process using a nested primer. The single chain product was then utilized in the hybridization reaction (pre-annealing) with the stacking oligonucleotides ^32^P labeled AUX-5 and AUX-3. Following a purification step, all the unincorporated auxiliary oligonucleotides were eliminated and the targets were ready for the final hybridization reaction with the capture probes.

Three cell lines were selected, HeLa, T18-3 and B18-3, for each of the mutations that belonged to one of the phylogenetic branches observed within HPV-18. Our results show that the B18-3 cell line was recognized by the probes P1 and P2, confirming that it belongs to the A-A variants (Figure 5H). Also, HeLa and T18-3 were detected for the EU and AF variants respectively (Figure 6I,J). Our microarray confirmed that punctual mutations in the cell line B18-3, belong to A-A variant. This contains the wild type, reference or prototype form of the polymorphism located at 7486, 7496 and 7529/7530 nt, which is the reference sequence related to the A-A branch (Figure 5J). Results from the HeLa cell line reveal that it possesses two punctual changes at 7529 nt (C→T) and 7486 (C→A). This confirms that HeLa cell line contains mutations related to the EU phylogenetic branch (Figure 5H). These results also illustrates that T18-3 cell line belonged to AF phylogenetic branch, the probe capture recognized two punctual site mutations the 7496 (C→G) and 7530 (T→C) present in the LCR (Figure 6I).

### 3.5. HPV-18 Positive Sample Hybridization

The aim of the current study was to develop and validate a simple, precise and accurate method to detect HPV-18 variants in CC patients. Single strand DNAs from 34 CC HPV-18 positive tissue samples were tested on our microarray. Representative patterns of hybridization for EU variant, AF variant and A-A variant are shown in Figure 5 hybridizations were as follows: (K) Hybridization pattern that corresponds to the A-A variant; (L) EU positive signal; (M) AF variant signal pattern. We considered the possibility of patients possessing multiple-variant infection affecting the sensitivity levels and specificity of the assay (which was not the case for single stDNA). Therefore, single target chains belonging to two out of three possible variants were pulled in pairs using all the three possible combinations (A-A/EU; EU/AF; AF/A-A), so each combination was hybridized on our microarray. In all cases, both variants gave positive signals, whereas the lacking third party was not detected. This suggests that there was no evidence of competition between them or any cross-reaction observed (data no shown). The samples were sequenced in order to validate our microarray results resulting a 100% of correlation between both techniques. Our results show that the WT sequence was detected in 22 of these 34 samples (64.7%). Nine DNA samples (26.47%) showed positive signals for the EU variant, indicating the presence of punctual mutations at position 7496 and 7529. Correspondently, only three samples were detected by the punctual mutation 7486 and 7530 which belong to the AF variant (Table 2).

Our phylogenetic analysis shows that the A-A variant is less closely related to its other two counterparts. In terms of its genomic identity, our data shows that there is a strong A-A component within our HPV-18 infected study population. Therefore, this data supports the fact that A-A variant is more frequent within Mexican population due to geographical distribution of HPV variants [11]. In addition, the complex genetic composition of the Mexican population might also explain why the rest of other variants (EU and AF) were also present as in some of the patients.

Recent works have contributed to achieve new CC etiologic evidences and promising HPV vaccination [36]. However, the epidemiological impact of the vaccination could be delay a few years until a significant decreasing of HPV infected incidence may occur, besides, a major percentage of the population worldwide is not getting vaccinated. In the meantime, oncogenicity might keep continue in a high frequency until we changes in mortality rates may diminished. Although array assays for the detection of HPV types, particularly high *vs.* low oncogenic risk are already available [37,38], these assay types cannot distinguish between different oncological behaviors.

High-risk HPVs are not restrictive from any particular histological type. Indeed, most of the HPV's high-risk detections would not progress to any stage of carcinoma. Because of that, it is necessary the identification of the viral variant and polymorphism, in order to make a better prognosis. As well as being faster and straightforward than other available assays; this new method's low cost and application is ideal for a large number of cases. This highly specific assay can be used to complement the diagnosis process. In consequence, it could be used for clinical implications with different HPV-18 variants. It is well documented that the infection with high-risk types HPVs are highly associated especially with rather more aggressive CC and in consequence many different molecular strategies have been designed to detect HPVs types [39].

Genomic variations in HPV types are not only restricted to coding genes as E2, E6 or E7, but also to non-coding regulatory sequences, such as the LCR region [11]. In the case of HPV-18, single base changes are located in defined spots of LCR, and could produce changes in its transcriptional properties. The HPV-18 variants are associated with 2 divergent phenotypes: (i) aggressive CC and a preponderance of cancer relative to cervical intra-epithelial neoplasia (CIN) and (ii) benign warty lesions of the cervix [40]. Several reports demonstrate that significant sequence variation within the E2 gene is associated with the presence of an HPV-18 subtype with decreased oncogenic potential [41,42]. Although, it is possible to consider these subtypes are not exclusive from any specific histological type but are specifically associated with certain histological types [42]. Non-European variants have been commonly associated with adenocarcinoma [43]. On the other hand, other works show that HPV-18 infections generally have a tendency to persist more frequently and to be more associated with pre-invasive lesions [17]. Therefore, early detection of HPV-18 variants is extremely important in order to reduce the incidence of pre-CC. Therefore, although little is known about the direct biological mechanism of the HPV variants, it is logical to assume that they could be relevant to the carcinogenic process.

When designing this assay, it was emphasized to create a low cost, affordable assay in terms of equipment and reagent availability for any laboratory with PCR facilities and utilizing just three probes to detect each phylogenetic branch in the same target, using only one PCR fragment. When compared with sequencing, this method is fast; the cost is considerably lower taking in to account that it can be reutilize several times showing without losing specificity. It is also worth mentioning that our design allowed the accurate detection of HPV-18 variants in CC samples, even in potentially multi-HPV-infected patients. In the case of sequencing method which represents a minority species in the total PCR product, it may underestimate the prevalence of infections with multiple HPV genotypes, with important consequences for follow-up studies [44]. Southern blot hybridization (SBH) and In situ hybridization can be used, but have serious limitations such as low sensitivity, time-consuming protocols, and the need for possibly large amounts of highly purified DNA. Future studies should be conducted to examine possible mechanisms involving variant-specific immune evasion and their potential clinical and therapeutic implications. The method described in the present work offers several advantages over common oligonucleotide array modes in which each target sequence consists of a single surface-tethered probe. The use of long stacking probe targets which reduces formation of secondary structures, results in increased sensitivity.

## 4. Conclusions

Our results show that the ability to detect HPV-18, prototype, variant 1 and variant 2 simultaneously, was possible due to the current state of the Genosensor system's technology [45]. It is also important to remark that the existing technology offers one of the highest specificities possible [46]. The PCR scheme and the oligonucleotide microarray described are effective tools to rapidly screen multiple virulence genes and their variant strains. Previous results demonstrate that SNP identification by oligonucleotide probe microarray was a useful technique for paternity testing and individual identification. These tools are also best suited for detecting the presence or absence of genetic sequences characteristic of specific pathogens, but microarrays are poorly suited for determining pathogen viability, and current methods provide only limited potential for pathogen enumeration. The feasibility and accuracy of the RT-PCR-RLB assay for detecting respiratory viruses proves that such an approach could be the first stage to develop a microarray assay for routine diagnosis of infectious diseases [47]. We are considering improving the current method by including more automatized techniques and isotope-free strategies. In this case we ignore at the moment if the non-radioactive labeling will affect the current sensitivity, which is crucial to discriminate among single nucleotide variants. In conclusion, our microarray analysis is confirmed as a viable model to detect all HPV-18 variants. However, this kind of technology needs to be tested massively with a view to clinical prognostic trials for CC.

## Figures and Tables

**Table 1.** Capture probes, stDNAs, stabilizers and primers employed for oligoarray development.

**Role**	**Sequence**	**Length (nt)**	**Name**
**Probe**	5′-ATGTCTGT-@	8	AA7486
	5′-ATGTTTGT-@	8	EU7486
	5′-TTTCTGCA-@	8	AA7496
	5′-TTTGTGCA-@	8	AF7496
	5′-TTGAAAT-@	7	AA7529/30
	5′-TTTAAAT-@	7	EU7529
	5′-TTGGAAT-@	7	AF7530

**Synthetic Target**	TTAGGAGGTAAAACGACACGTTGGCTAAAGC CAACGGAAACCGAATACAGACACCAAAAGAC GTGTTATG	92	STAA7486
	TTAGGAGGTAAAACGACACGTTGGCTAAAGC CAACGGAAACCGAATACAAACACCAAAAGAC GTGTTATG	92	STAF7486
	TTAGGAGGTAAAACGACACGTTGGCTAAAGC CAACGGAAACCGAATACAAACACCAAAAGAC GTGTTATG	92	STAA7496
	TTAGGAGGTAAAACGACACGTTGGCTAAAGC CAACGGAAACCGAATACAAACACCAAAACAC GTGTTATG	92	STEU7496
	GCACAATACAGTACACTGGCACTATTGCAAA CTTTAATCTTTTGGGCACTGCTCCTAC	58	STAA7529/30
	GCACAATACAGTACACTGGCACTATTGCAAA ATTTAATCTTTTGGGCACTGCTCCTAC	58	STEU7529
	GCACAATACAGTACACTGGCACTATTGCAAAC CTTAATCTTTTGGGCACTGCTCCTAC	58	STAF7530

**Stabilizer**	AATCCTCCATTTTGCTGTCAACCGATTTCG GTTGCCTTTGGCTT	45	AUX5′7486
	GGTTTTCTGCACAATACAGTACACTGGCACT ATTGCAAA	39	AUX3′7486
	AATCCTCCATTTTGCTGTCAACCGATTTCG GTTGCCTTTGGCTTATGTCTGTGGT	56	AUX5′7496
	CAATACAGTACACTGGCACTATTGCAAA	28	AUX3′7496
	CGTGTTATGTCATGTGACCGTGATAACG	28	AUX5′7529/30
	TAGAAAACCCGTGACGAGGATG	22	AUX3′7529/30

**Primer**	GTAGGAGCAGTGCCCAAAAG	20	ds Primer 3′
	AATCCTCCATTTTGCTGTGC	20	ds Primer 5′
	GCACAATACAGTACACTGGCACT	23	ss Primer 5′
	TTTGCAATAGTGCCAGTG	18	ss Primer 5′

**Table 2.** Positive signal patterns for CC samples showing variant specificity.

CC (n)	Positive AA7486	Hybridization AF7486	Signal AA7496	Pattern EU7496	AA7529/30	EU7529	AF7530	Variant	%
**22**	X		X		X			AA	64.70
**9**				X		X		EU	26.47
**3**		X					X	AF	8.82
**34**									99.99
